# Recombinant Humanized IgG1 Antibody Promotes Reverse Cholesterol Transport through FcRn-ERK1/2-PPARα Pathway in Hepatocytes

**DOI:** 10.3390/ijms232314607

**Published:** 2022-11-23

**Authors:** Zhonghao Li, Qi Zhang, Xianyan Liu, Ming Zhao

**Affiliations:** 1 Key Lab for Shock and Microcirculation Research of Guangdong, Department of Pathophysiology, School of Basic Medical Sciences, Southern Medical University, Guangzhou 510515, China; 2 The First School of Clinical Medicine, Southern Medical University, Guangzhou 510515, China

**Keywords:** hyperlipidemia, recombinant humanized IgG1 antibody, reverse cholesterol transport, hepatocytes

## Abstract

Hyperlipidemia-associated lipid disorders are considered the cause of atherosclerotic cardiovascular disease. Reverse cholesterol transport (RCT) is a mechanism by which excess peripheral cholesterol is transported to the liver and further converted into bile acid for excretion from the body in feces, which contributes to reducing hyperlipidemia as well as cardiovascular disease. We previously found that the recombinant humanized IgG1 antibody promotes macrophages to engulf lipids and increases cholesterol efflux to high-density lipoprotein (HDL) through ATP-binding cassette sub-family A1 (ABCA1), one of the key proteins related to RCT. In the present study, we explored other RCT related proteins expression on hepatocytes, including scavenger receptor class B type I (SR-BI), apolipoprotein A-I (ApoA-I), and apolipoprotein A-II (ApoA-II), and its modulation mechanism involved. We confirmed that the recombinant humanized IgG1 antibody selectively activated ERK1/2 to upregulate SR-BI, ApoA-I, and ApoA-II expression in mice liver and human hepatocellular carcinoma cell lines HepG2 cells. The rate-limiting enzymes of bile acid synthesis, including cholesterol 7α-hydroxylase (CYP7A1) and sterol 27-hydroxylase (CYP27A1), exhibited a significant increase when treated with the recombinant humanized IgG1 antibody, as well as increased excretion of bile acids in feces. Besides, abolishment or mutation of peroxisome proliferator-activated receptor α (PPARα)/RXR binding site on SR-BI promoter eliminated SR-BI reporter gene luciferase activity even in the presence of the recombinant humanized IgG1 antibody. Knock down the neonatal Fc receptor (FcRn) on hepatocytes impaired the effect of recombinant humanized IgG1 antibody on activation of ERK1/2, as well as upregulation of SR-BI, ApoA-I, and ApoA-II expression. In conclusion, one of the mechanisms on the recombinant humanized IgG1 antibody attenuates hyperlipidemia in ApoE^−/−^ mice model fed with high-fat-diet might be through reinforcement of liver RCT function in an FcRn-ERK1/2-PPARα dependent manner.

## 1. Introduction

Dyslipidemia is characterized by increased blood levels of total or LDL cholesterol and triglycerides with decreased HDL cholesterol levels and is a risk factor for cardiovascular diseases such as atherosclerosis [1,2]. With its high prevalence worldwide, dyslipidemia is a major public health challenge due to its extremely high morbidity. Many patients are turning to alternatives to pharmacotherapy to manage their lipid levels. In the present study, a generally recognized atherosclerosis model involving ApoE^−/−^ mice fed with a high-fat diet (HFD) was applied to mimic the situation of patients with atherosclerotic heart disease suffering from hyperlipidemia in the clinic. Consistent with previous reports, ApoE^−/−^ mice fed with high-fat diets are susceptible to hyperlipidemia [3].

Reverse cholesterol transport (RCT) removes excess cholesterol from peripheral cells and tissues, delivering the cholesterol to hepatocytes for excretion. ATP-binding cassette transporter A1 (ABCA1) mediated cholesterol efflux from peripheral cells to form HDL particles is the initial step of RCT. Subsequently, cholesterol-loaded HDL is transferred to hepatocytes and taken up by hepatocytes, then converted into bile, and finally excreted in the feces [4]. Scavenger receptor class B type I (SR-BI) is a physiological high-affinity receptor for HDL, mediates the selective uptake of lipids from HDL to hepatocytes, and thus plays a crucial role in the later stage of RCT [5,6].SR-BI has been acknowledged as a potential new target for cardiovascular drugs. Therefore, enhancement of RCT is beneficial to reduce atherosclerotic lesions by accelerating the clearance of excess cholesterol in extrahepatic tissue cells and promoting cholesterol reduction in early atherosclerotic lesions. Thus, drugs that increase SR-BI expression can limit the occurrence of hyperlipidemia by accelerating the RCT process and promoting the elimination of excess lipid accumulation.

Our previously experimental data have confirmed that the recombinant humanized IgG1 antibody maintains liver triglyceride homeostasis through arylacetamide deacetylase [7]. Recently, we found that the antibody promotes macrophages to engulf lipids and efflux to HDL through ABCA1 (data not shown), one of the key proteins in RCT. These results show that the recombinant humanized IgG1 antibody might have the function of reinforcing RCT in response to lipid metabolism disorders. Considering that the ERK1/2-proliferator-activated receptor α (PPARα) pathway is involved in the modulation of SR-BI expression and cholesterol homeostasis [8], we hypothesized that the recombinant humanized IgG1 antibody might impede dyslipidemia by enhancing hepatic RCT in an FcRn-ERK1/2-PPARα pathway-dependent manner.

## 2. Results

### 2.1. The Antibody Inhibits the Development of Hyperlipidemia in ApoE^−/−^ Mice

**Figure 1:**
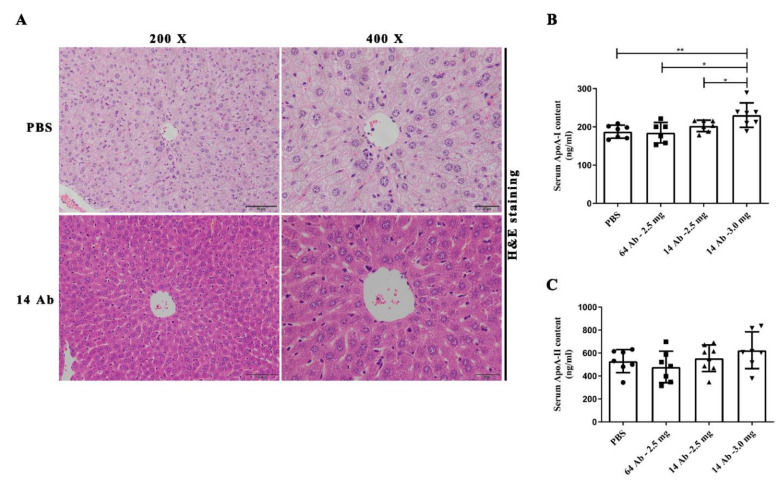
The antibody improves steatosis in mice livers and increases serum ApoA-I content. (**A**) H&E staining of liver sections from ApoE^−/−^ mice treated with PBS or 14 Ab. (**B**,**C**) The quantitative determination of mouse ApoA-I and ApoA-II in serum by ELISA, respectively. Data are shown as mean ± SD (n = 7). *, *p* < 0.05; **, *p* < 0.01.

To examine the anti-hyperlipidemia effect of the recombinant humanized IgG1 antibody, the content of total cholesterol, triglyceride, HDL-C, and LDL-C in the liver was detected. As shown in Table 1, the level of triglyceride and LDL-C were decreased with increased HDL-C levels in mice liver after 14 Ab administration compared with PBS. Another clone of the antibody, 6 Ab, also showed a decrease in triglyceride. Unexpectedly, the decreased triglyceride and LDL-C with increased HDL-C were also observed in the combination of 14 Ab and 6 Ab. These results indicate that the antibody improves HFD-induced lipid abnormalities in atherosclerosis-prone mice. ApoE^−/−^ mice fed with HFD not only suffer from hyperlipidemia but also induce liver steatosis [9,10,11]. To confirm whether the antibody has a protective effect in the liver, liver steatosis was determined in ApoE^−/−^ mice. As shown in Figure 1A, in contrast to PBS administration, the HFD-induced hepatic steatosis was significantly attenuated in 14 Ab-treated mice by H&E staining. These observations demonstrate the critical role of the antibody in liver protection. At the same time, we collected serum to determine the content of serum ApoA-I and ApoA-II. ApoA-I was significantly increased in 14 Ab (3.0 mg)-treated mice, but no difference was observed in ApoA-II (Figure 1B,C).

### 2.2. The Antibody Promotes Liver Reverse Cholesterol Transport in ApoE^−/−^ Mice

**Figure 2:**
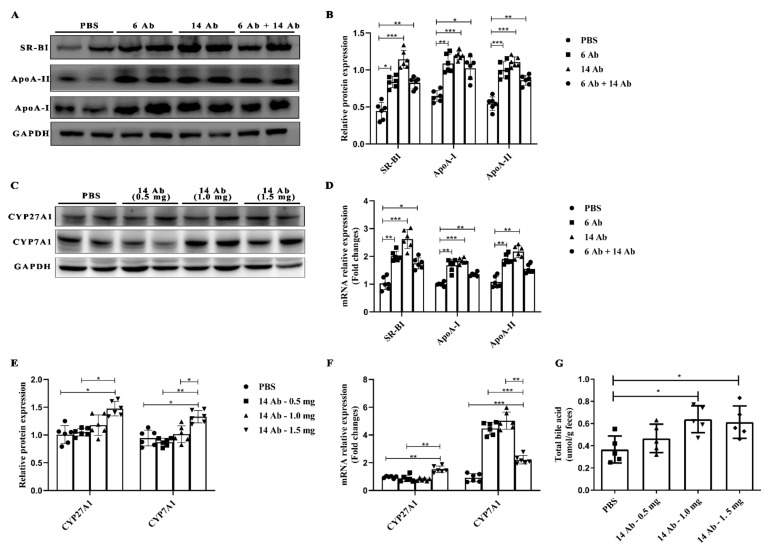
The antibody promotes liver reverse cholesterol transport in ApoE^−/−^ mice. (**A**) The protein expression of SR-BI, ApoA-II, and ApoA-I in ApoE^−/−^ mice liver was determined by Western blot, GAPDH was used as an internal control (n = 6); (**B**,**D**) Gray value analysis of protein expression and mRNA transcription of SR-BI, ApoA-II and ApoA-I in mice liver, respectively; (**C**) The protein expression of CYP7A1 and CYP27A1 in ApoE^−/−^ mice liver was determined by Western blot, GAPDH was used as an internal control (n = 6); (**E**,**F**) Gray value analysis of protein expression and mRNA transcription of CYP7A1 and CYP27A1 in mice liver, respectively; (**G**) Detection of bile acid content in mouse feces (n = 5). Data are shown as mean ± SD. *, *p* < 0.05; **, *p* < 0.01; ***, *p* < 0.001.

The reverse cholesterol transport pathway involves several critical proteins, including ABCA1, ApoA-I, ApoA-, and SR-BI. To determine the role of the antibody in regulating other RCT related proteins expression in the liver, such as ApoA-I, ApoA-II, and SR-BI, ApoE^−/−^ mice were fed with HFD for 20 weeks and then treated with the antibody or PBS. The antibody administration, either 6 Ab or 14 Ab alone or in combination, exhibited significant upregulation of ApoA-I, ApoA-II, and SR-BI expression in both protein and mRNA levels, compared to PBS administration (Figure 2A,B,D). Especially, it is observed that 14 Ab works best. Next, we investigated whether the 14 Ab administration increases bile acid synthesis.

**Figure 3:**
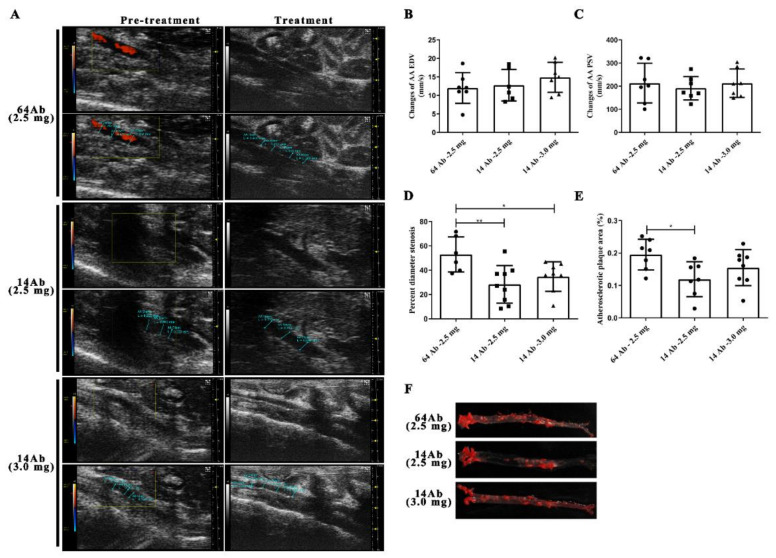
The antibody improves HFD-induced aorta atherosclerosis in ApoE^−/−^ mice. (**A**) Vevo 2100 ultrasound imaging system examination of the abdominal aorta inner diameter in mice. (**B**–**D**) The abdominal aorta peak systolic velocity, abdominal aorta end diastolic velocity, and abdominal aorta diameter stenosis were measured, respectively. (**E**) Plaque areas in the aortas were assessed by the enface Oil Red O staining, and the percentage-stained area of the total aortic area was determined by Image Pro Plus. (**F**) Representative image of Oil red O staining of plaque in the aorta, plaque is indicated with red color. Data are shown as mean ± SD. *, *p* < 0.05, **, *p* < 0.01.

Bile acid biosynthesis is involved in two major pathways, namely the classic pathway and the alternative pathway [12]. The classic pathway is initiated by cholesterol 7α-hydroxylase (CYP7A1) located in the endoplasmic reticulum of hepatocytes, whereas the alternative pathway is initiated by mitochondrial sterol 27-hydroxylase (CYP27A1). CYP7A1 and CYP27A1 are the two rate-limiting enzymes in bile acid synthesis [13]. Thus, we measured the expression of CYP7A1 and CYP27A1 in the liver of mice subjected to different doses of treatment with 14 Ab. Both CYP7A1 and CYP27A1 protein expression rose significantly in 14 Ab (1.5 mg), while 14 Ab (1.0 mg) got only a slight increase compared to the PBS group, and no differences were observed in 14 Ab (0.5 mg) (Figure 2C,E). Consistent with protein expression, mRNA transcription of CYP27A1 and CYP7A1 in the liver showed significant upregulation in 14 Ab (1.5 mg) (Figure 3F). Next, we investigated whether the antibody participates in bile acid excretion. The results demonstrated that the total bile acids content in feces was increased upon administration of 14 Ab (both 1.0 mg and 1.5 mg) (Figure 2G). These results indicate that the antibody promotes liver reverse cholesterol transport.

### 2.3. The Antibody Improves HFD-Induced Aorta Atherosclerosis

We previously reported that the recombinant humanized IgG1 antibody reduces atherosclerosis [14]. To determine the histological changes in the abdominal aorta, ApoE^−/−^ mice were subjected to the Vevo 2100 Ultrasound Imaging System before and after the antibody treatment. There were no significant differences in abdominal aorta luminal diameter before the antibody treatment, whereas 14 Ab administration (2.5 mg and 3.0 mg) was larger compared to 64 Ab, the control isotype antibody (Figure 3A and Table 2). Surprisingly, diameter stenosis in 14 Ab (both 2.5 mg and 3.0 mg) was significantly smaller than that in 64 Ab, while 14 Ab (2.5 mg) had the best effect (Figure 3D). Similarly, 14 Ab (2.5 mg) treatment resulted in a more than 38% regression of atherosclerosis in the aorta by en face Oil Red O staining compared to 64 Ab treatment (Figure 3E,F). However, we found that the abdominal aorta peak systolic velocity was not affected as well as the abdominal aorta end diastolic velocity before and after antibody treatment (Figure 3B,C).

### 2.4. The Antibody Upregulates the Expression of SR-BI, ApoA-II, and ApoA-I in HepG2 Cells

**Figure 4:**
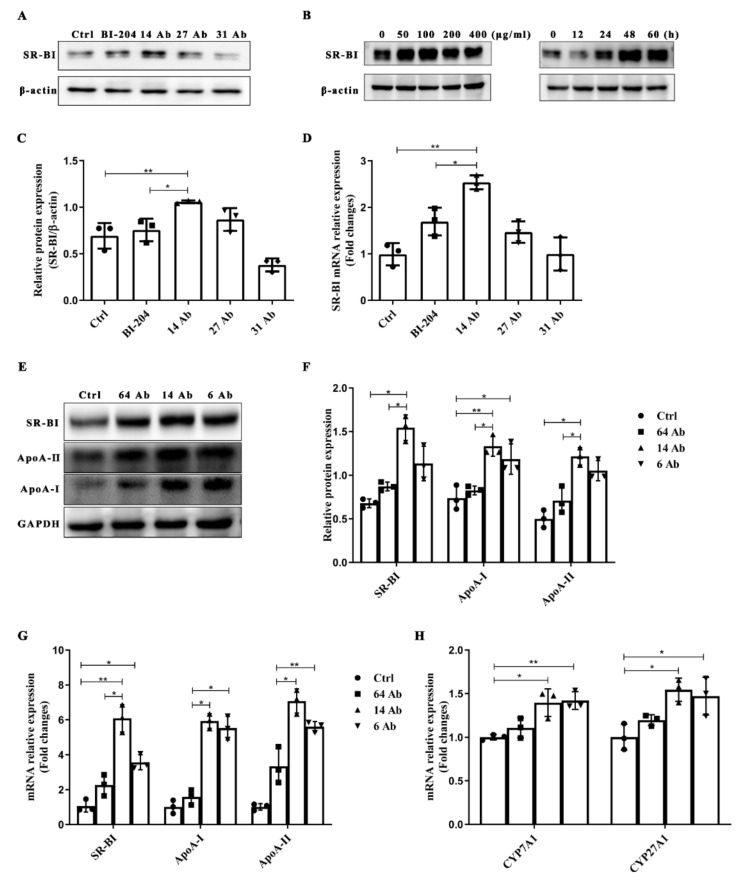
The antibody enhances RCT-related protein expression in HepG2 cells. (**A**) HepG2 cells were treated with the antibody (14 Ab, 27 Ab, 31 Ab) at 50 µg/mL for 24 h; BI-204 is an ox-LDL antibody. Western blotting was used to determine the protein expression of SR-BI, β-actin was used as an internal control (n = 3); (**C**) Gray value analysis and (**D**) RT-qPCR was applied to measure mRNA transcription of SR-BI. (**B**) SR-BI protein expression in different doses and increasing incubated time of 14 Ab, β-actin was used as an internal control (n = 3). (**E**,**F**) HepG2 cells were treated with the antibody at 100 µg/mL for 24 h; Western blotting was used to determine the protein expression of SR-BI, ApoA-II, and ApoA-I and gray value analysis, respectively. GAPDH was used as an internal control (n = 3). (**G**,**H**) The mRNA transcription of SR-BI, ApoA-I, ApoA-II, CYP7A1 and CYP27A1 was measured, respectively (n = 3). Data are shown as mean ± SD. *, *p* < 0.05; **, *p* < 0.01.

Increased expression of ApoA-I, ApoA-II, and SR-BI has previously been shown in the liver of ApoE^−/−^ mice treated with the antibody to further investigate the signal transduction pathway in the antibody-induced genes expression involved in RCT in hepatocytes, protein expression and mRNA transcription of ApoA-I, ApoA-II and SR-BI were firstly determined in HepG2 cells. As shown in Figure 4A,C,D, 14 Ab potently amplified the expression of SR-BI both at the mRNA transcription and protein levels compared to BI-204, an ox-LDL monoclonal antibody provided by Jan Nilsson Laboratory (Lund University, Sweden). To further verify the optimal working condition of the antibody, HepG2 cells were incubated with 14 Ab at different concentrations (0, 50, 100, 200, and 300 μg/mL, respectively) for different durations (0, 12, 24, 48, 60 h, respectively). Then SR-BI protein levels were visualized by western blot analyses. The results showed that 14 Ab potently upregulated the protein expression of SR-BI in a concentration- and time-dependent manner (Figure 4B). We then chose the antibody incubated for 24 h at 100 μg/mL as the subsequent working conditions. As shown in Figure 4E,F, incubation with 14 Ab dramatically upregulated the protein expression of ApoA-I, ApoA-II, and SR-BI, while no differences were seen in 64 Ab, the control antibody. These results were also confirmed at the mRNA transcriptive level (Figure 4G). Moreover, we observed that 14 Ab treatment also increased the mRNA transcription of the two rate-limiting enzymes in bile acid synthesis, namely CYP7A1 and CYP27A1 (Figure 4G).

### 2.5. The Antibody Upregulates the Expression of ApoA-I, ApoA-II and SR-BI Is ERK1/2 Dependent

**Figure 5:**
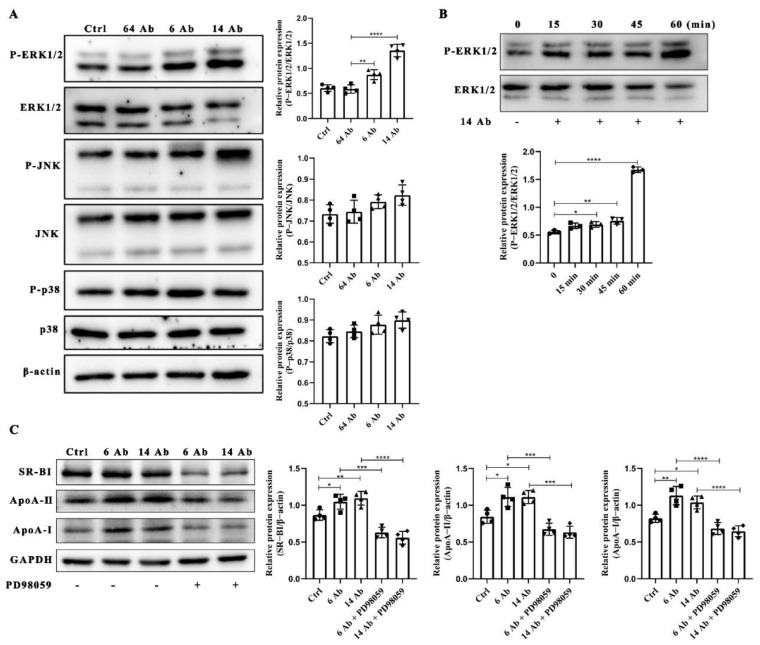
The antibody upregulates SR-BI, ApoA-I, and ApoA-II protein expression through activation of ERK1/2. (**A**) HepG2 cells were treated with the antibody at 100 μg/mL for 60 min, and the proteins were collected from cell lysates and subjected to Western blot to determine the activation of JNK, ERK1/2, and p38 (n = 4). (**B**) HepG2 cells were treated with 14 Ab at 100 μg/mL in increasing incubated time to measure ERK1/2 activation (n = 3). (**C**) HepG2 cells were pretreated with ERK1/2 inhibitor PD98059 (10 μM) for 30 min, following incubated with the antibody for another 24 h to determine the protein expression of SR-BI, ApoA-I, and ApoA-II (n = 4). Data are shown as mean ± SD. *, *p* < 0.05; **, *p* < 0.01; ***, *p* < 0.001, ****, *p* < 0.0001.

HepG2 cells were starved in 1% FBS-containing medium for 8 h, following treated with the antibody at 100 μg/mL for 60 min. Data showed that the antibody (6 Ab and 14 Ab) specifically activates ERK1/2 but not p38 and JNK (Figure 5A). Moreover, we found 14 Ab-induced activation of ERK1/2 in a time-dependent manner (Figure 5B). At the same time, we verified deactivation of ERK1/2 caused by PD98059 impaired the upregulation of ApoA-I, ApoA-II, and SR-BI expression even in the presence of the antibody (Figure 5C). These results indicate that the antibody-induced upregulation of ApoA-I, ApoA-II, and SR-BI expression is ERK1/2 dependent.

### 2.6. The Antibody Upregulates SR-BI, ApoA-I, and ApoA-II Protein Expression through FcRn

**Figure 6:**
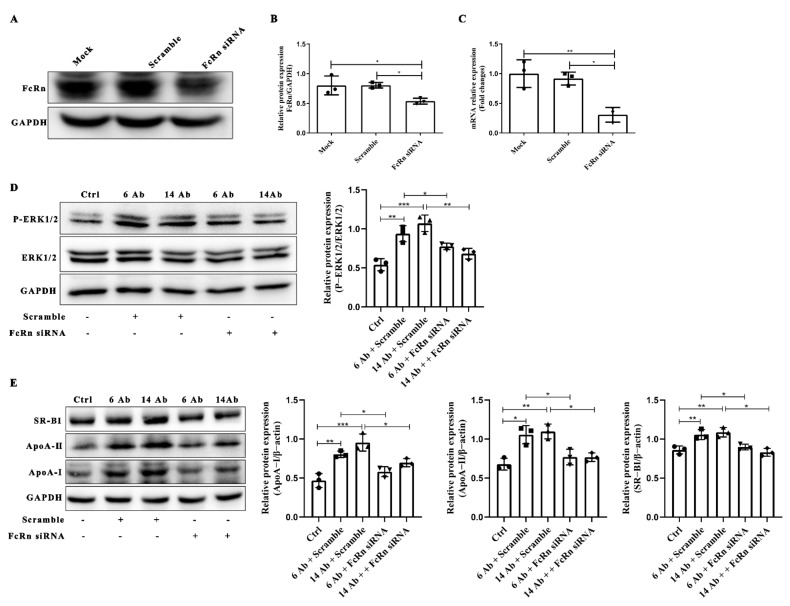
The antibody upregulates SR-BI, ApoA-I, and ApoA-II protein expression through FcRn. HepG2 cells were transfected with FcRn siRNA or scramble for 48 h, and the protein expression (**A**,**B**) and mRNA level (**C**) were measured (n = 3). (**D**) HepG2 cells were transfected with FcRn siRNA or scramble for 48 h, following incubation with the antibody for another 60 min to determine the activation of ERK1/2 (n = 3). (**E**) HepG2 cells were transfected with FcRn siRNA or scramble for 48 h, following incubated with the antibody for another 24 h to determine the protein expression of SR-BI, ApoA-I, and ApoA-II (n = 3). Data are shown as mean ± SD (n = 3). *, *p* < 0.05; **, *p* < 0.01; ***, *p* < 0.001.

The neonatal FC receptor (FcRn) is a specific IgG receptor involved in the transcellular transport of IgG, and we previously demonstrated that FcRn is responsible for the regulation of arylacetamide deacetylase expression in the presence of recombinant humanized IgG1 [7]. To determine whether FcRn is responsible for the upregulation expression of ApoA-I, ApoA-II, and SR-BI induced by the antibody, we first knockdown endogenous FcRn in HepG2 cells by FcRn-specific siRNA (Figure 6A−C). Thereafter, HepG2 cells were transfected with FcRn siRNA for 48 h, followed by incubation with the antibody for 60 min to determine the expression of ApoA-I, ApoA-II, and SR-BI. As shown in Figure 6D, siRNA-mediated knockdown of endogenous FcRn almost abolished the activation of ERK1/2 induced by the antibody. At the same time, in the absence of FcRn, the increased expression of ApoA-I, ApoA-II, and SR-BI induced by the antibody was blocked (Figure 6E).

### 2.7. The Antibody Upregulates the Expression of SR-BI Is Regulated by PPARα

**Figure 7:**
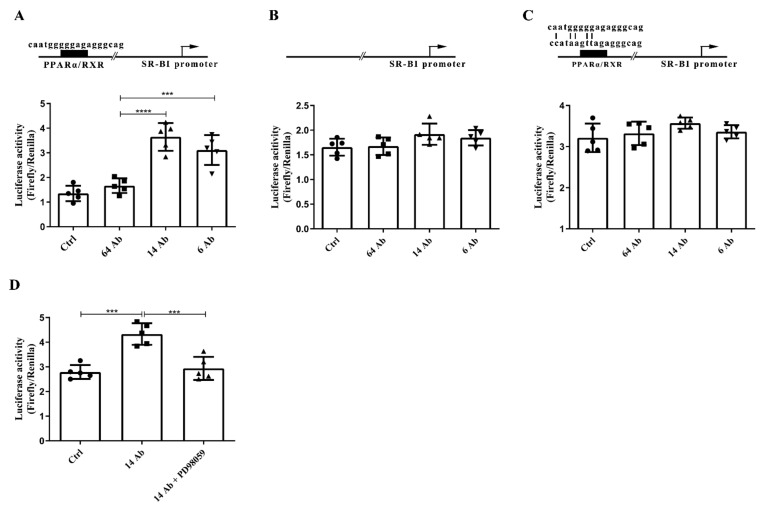
The antibody upregulated the expression of SR-BI and is regulated by PPARα. HepG2 cells were transfected with the plasmid of (**A**) SR-BI promoter (−2500~+21 bp)-Luciferase and (**B**) SR-BI promoter truncation (−2480~+21 bp)-Luciferase and (**C**) SR-BI promoter mutant (−2500~+21 bp)-Luciferase. The PRL-TK plasmid was co-transfected as a transfection control, following treated with the antibody for 24 h. Promoter activity was determined by relative luciferase activity, with the level normalized to that of Renilla (n = 5). (**D**) HepG2 cells were transfected with the plasmid of SR-BI promoter (−2500~+21 bp)-Luciferase and PRL-TK plasmid for 24 h, then treated with or without PD98059 (10 μM) for 30 min, following incubated with the antibody for another 24 h to determine the luciferase activity. Data are shown as mean ± SD (n = 5). ***, *p* < 0.001; ****, *p* < 0.0001.

It is reported that PPARα modulates the key protein expression associated with reverse cholesterol transport, such as SR-BI [15], ApoA-I [16], and ApoA-II [17], a process that removes peripheral excess lipids. Since PPARα is abundantly expressed in the liver and brown adipose tissue [18] and SR-BI serves as a receptor of HDL, we pick up SR-BI expression as a sample model in this paper. To investigate whether PPARα is the key transcription factor in the antibody regulation of RCT-related protein expression, we constructed the SR-BI luciferase reporter gene containing PPARα/RXRα binding site and confirmed that a remarkable increase of SR-BI luciferase activity upon 14 Ab treatment compared to the control antibody, 64 Ab (Figure 7A). Furthermore, when we mutated or truncated PPARα/RXRα binding site, the antibody treatment did not have any effect on these SR-BI reporter luciferase activity anymore (Figure 7B,C). These results demonstrated that the antibody upregulates the expression of SR-BI through transcription factor PPARα.

### 2.8. The Antibody Promotes Dil-HDL Uptake Is ERK1/2 Dependent

**Figure 8:**
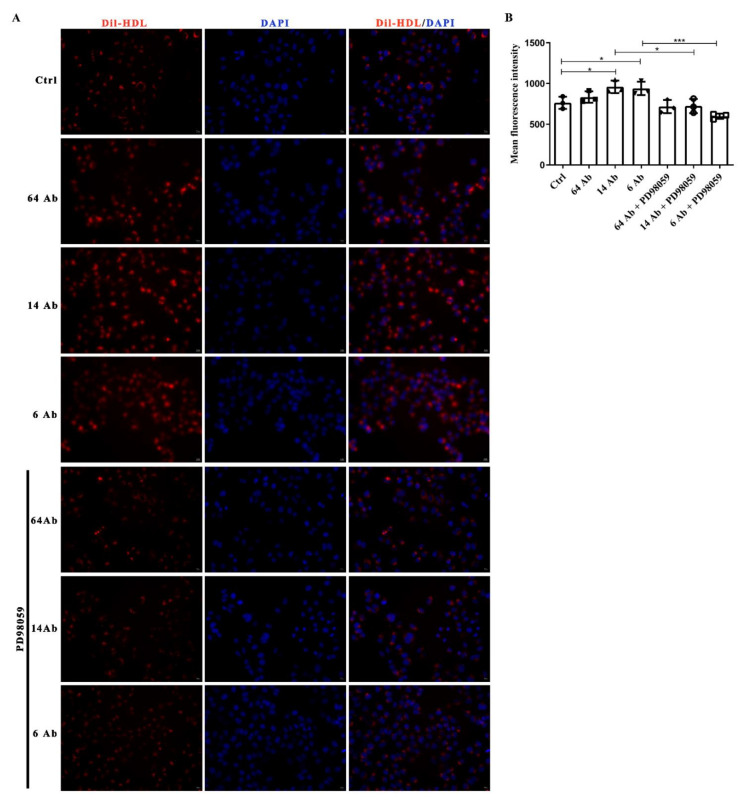
The antibody increases the uptake of Dil-HDL, while PD98059 inhibits it. (**A**) HepG2 cells were pretreated with or without PD98059 (10 µM) for 30 min, then treated with the antibody (100 µg/mL) for 24 h and subsequently incubated with Dil-HDL (10 µg/mL) for an additional 4 h at 37 °C. After stimulation, the cells were fixed with 4% paraformaldehyde and incubated with DAPI. Images were taken using a ZESIS fluorescent microscope. (**B**) The relative fluorescence intensity of the uptake of Dil-HDL was determined by ZEN 3.4 software. Data are shown as mean ± SD (n = 3). *, *p* < 0.05; ***, *p* < 0.001.

To confirm the importance of ERK1/2 activation on the antibody-induced upregulation of HDL receptor, SR-BI, Dil-HDL binding assay was performed to further determine cholesterol uptake in HepG2 cells. Incubation with the antibody for 24 h resulted in a modest but significant increment of Dil-HDL uptake in HepG2 cells, but no significant difference was observed in the presence of ERK1/2 inhibitor PD98059 (Figure 8A,B).

## 3. Discussion

Hyperlipidemia has been ranked as one of the greatest risk factors contributing to the prevalence and severity of coronary heart disease. Hyperlipidemia-associated lipid disorders are considered the cause of atherosclerotic cardiovascular disease [19]. Despite the development in treating patients with pathological hyperlipidemia, imbalanced lipid homeostasis exacerbates atherosclerotic heart diseases and remains a serious problem in cardiovascular studies. Considerable evidence has demonstrated that the enhancement of RCT and the accompanying increase in HDL levels play essential roles in preventing hyperlipidemia development [20]. Healing after high-fat diet-induced hyperlipidemia provides a therapeutic possibility for attenuating hyperlipidemia. In this paper, we described a potential therapeutic effect of the recombinant humanized antibody, which can attenuate high-fat diet-induced hyperlipidemia via enhancement of RCT in atherosclerosis-prone mice.

Hyperlipidemia is a multifactorial clinical syndrome induced by a large number of physiological stimuli and pathological insults, including a diet high in saturated or trans fats, physical inactivity, smoking, and obesity [21]. Several genetic factors, especially in the gene-encoding enzymes characterized by the RCT system, are responsible for reversing hyperlipidemia incidence. Statins are the main treatment for hyperlipidemia [22]; however, the limitations of statins include treatment resistance, intolerance due to adverse events, and a lack of adherence which contribute to poor outcomes. As such, many patients require adjunct therapies to properly control hyperlipidemia, including niacin, bile acid sequestrants, fibric acids, and cholesterol absorption inhibitor ezetimibe, as well as the recently approved class PCSK9 inhibitors. None of these are excellent treatments, with different application limitations. Thus, we develop an antibody therapy to overcome these limitations. However, the recombinant humanized antibody administration revealed remarkable advantages in alleviating the detrimental effects of abnormal lipid homeostasis disease in atherosclerosis-prone mice.

ABCA1-mediated cholesterol efflux from lipid-laden macrophages to ApoA-I is the first step of RCT, resulting in nascent HDL formation. Then apolipoprotein ApoA-II binds to the nascent HDL to form mature HDL. Finally, circular spherical HDL delivers its cholesterol cargo to the liver for further metabolism and secretion. This process is dependent on SR-BI-mediated selective HDL-C uptake in the liver. Accordingly, hepatic SR-BI is critical for plasma HDL-C clearance. It has been demonstrated that SR-BI binds HDL with high affinity and mediates the selective uptake of cholesteryl ester into the liver [5]. SR-BI also mediates the bidirectional flux of free cholesterol between cells and HDL [23,24]. The influx of HDL cholesteryl ester and free cholesterol by hepatic SR-BI and subsequent routing to bile is a major route of delivery of peripheral cholesterol to the liver for excretion in both mice and humans [25]. By mediating the uptake of HDL lipids, SR-BI preserves HDL function [26]. Our results show that the recombinant humanized antibody could upregulate SR-BI as well as ApoA-I and ApoA-II expression in the mice liver and HepG2 cells. Meanwhile, we demonstrate that the antibody could also increase the selective uptake of DiI-HDL in HepG2 cells. It has been reported that treatment with PPARα agonist Wy-14643 results in a pronounced induction of SR-BI expression [15,27] while blocking the activation of PPARα with PPARα antagonist GW6471 abolished the SR-BI expression [28]. Similarly, our results confirm that PPARα/RXR binds the SR-BI gene promoter and is implicated in SR-BI magnification induced by the antibody by using human SR-BI promoter-luciferase reporter gene construct and its mutants. The ERK1/2 signaling pathway has been implicated in modulating the activity of nuclear receptors, including peroxisome proliferator activator receptors α [8] and liver X receptors [29], to alter the ability of cells to transport cholesterol. Surprisingly, our data show that the antibody selectively activates ERK1/2 in HepG2 cells, which has no effect on JNK and p38, resulting in upregulation of SR-BI, ApoA-I, and ApoA-II expression. This is also coincident with the effect of ERK1/2 inhibitor on the suppression of antibody-induced DiI-HDL uptake. Although we found that the antibody regulates SR-BI expression through ERK1/2-PPARα/RXR signaling pathway, the more detailed molecular mechanism still needs to be explored further.

Bile acid synthesis is the predominant metabolic pathway for the catabolism of cholesterol. The bile acid biosynthetic pathway involves two pathways: classic bile acid biosynthetic pathway and alternative pathway, regulated by the rate-limiting enzyme CYP7A1 and CYP27A1, respectively. Here, we present that the antibody enhances CYP7A1 and CYP27A1 expression and ensures fecal bile acid excretion. These results extend our previous findings of the increased ABCA1 expression and cholesterol efflux machinery in macrophages and indicate a major role for the antibody as a regulator of cholesterol homeostasis and RCT. Although FcRn expression was identified in hepatocytes in 1995 by Blumberg and colleagues [30], the function of this receptor in hepatocytes has to date, not been fully elucidated. In the present study, we reported that the antibody presented its effect on hepatocytes depending on FcRn. However, what downstream molecule for FcRn could specifically activate ERK1/2 activity is still unclear. The detailed molecular mechanism of FcRn in signal transduction needs to be further explored.

In summary, the present study demonstrated that the recombinant humanized IgG1 antibody alleviates hyperlipidemia and in vitro study shows its lipid-lowering activity might be through reinforcement of RCT in an FcRn-ERK1/2-PPARα dependent manner.

## 4. Materials and Methods

### 4.1. Materials

Dulbecco’s modified Eagle’s medium (DMEM), RPMI 1640 medium, fetal bovine serum (FBS), phosphate-buffered saline (PBS), and HEPES were purchased from Invitrogen (Carlsbad, CA, USA). The TRIzol Reagent was from Takara Bio Inc. (Kusatsu, Japan). Antibodies recognizing phosphorylation of ERK1/2, p38, and JNK were from Cell Signaling Technology (Danvers, MA, USA). The anti-SR-BI antibody was from Novus Biologicals. The inhibitor of ERK (PD98059) was from Beyotime (Beijing, China). Antibodies against FcRn, ApoA-I, ApoA-II, and PPARα were purchased from Proteintech Group, Inc. (Rosemont, IL, USA). Dil-HDL was obtained from Yiyuan Biotechnologies (Guangzhou, China). FcRn siRNA was designed and synthesized by GenePharma (Suzhou, China). The recombinant humanized IgG1 antibody (clone NO.6, 14, 27, and 64, namely 6 Ab, 14 Ab, 27 Ab, and 64 Ab) was produced by our laboratory; the effectiveness of 14 Ab had been confirmed in our previous work [7,14]. In this paper, we mainly focus on 6 Ab and 14 Ab, and 64 Ab was an isotype-matched control IgG1 antibody [14].

### 4.2. Cell Culture

Human hepatoma cell line HepG2 was obtained from ATCC (Manassas, VI, USA) and was grown in DMEM supplemented with 10% FBS under standard culture conditions (5% CO_2_, 37 °C). All cells were maintained at 37 °C in 5% CO_2_ and 95% air.

### 4.3. RNA Isolation and RT-PCR Analysis

RT-PCR analysis was conducted as described previously [31]. Briefly, TRIzol reagent was used to extract total RNA in accordance with the manufacturer’s instructions. The cDNA was obtained by reverse transcription using First-Strand Cdna Synthesis Kit (Genecopoeia, Guangzhou, China), after which PCR was performed on a real-time RT-PCR machine (ABI 7500, USA). GADPH was used as an internal control. The sequences of the primers used for the experiment were shown in the Supplementary Materials Table S1. A single DNA duplex was generated by melting curve analyses, and the 2^−ΔΔCt^ method was used to perform quantitative measurements.

### 4.4. Western Blot Analysis

Western blot analysis was conducted as described previously [32]. Briefly, the total proteins were extracted, after which their concentrations were determined using a BCA kit (Beyotime; Beijing, China). The proteins (20~80 μg per lane) were subsequently separated by 10% SDS-PAGE and transferred to a PVDF membrane, immuno-blotted with antibodies against GAPDH, ApoA-I, ApoA-II, and SR-BI, respectively. Following a series of rinses in TBS-T, the membrane was incubated with a peroxidase-conjugated secondary antibody. Lastly, the proteins were visualized by enhanced chemiluminescence (ECL; Merck Millipore, Darmstadt, Germany), and the relative expression levels were assessed by Image J via densitometry.

### 4.5. Cell Transfection

HepG2 cells, when reached approximately 80% confluence, were transfected with small-interfering RNA (siRNA) specific for human FcRn using Lipofectamine^TM^ 2000 (Invitrogen) according to the instructions of manufacturers. After transfection for 48 h, cells were harvested for RT-qPCR and western blot analysis to evaluate the silencing efficiency.

### 4.6. Luciferase Reporter Gene Assays

A 2521bp fragment of human SR-BI promoter was amplified by PCR from the genome of the HepG2 cells and then cloned into the reporter vector pGL3-basic (Promega, Madison, WI, USA) and truncated SR-BI-Luc vector and mutated SR-BI-Luc vector were constructed by the same strategy. Transfection experiments were carried out in 24-well plates using Lipofectamine^TM^ 2000 (Invitrogen). 24 h after transfection, HepG2 cells were treated with or without the antibody for another 24 h. A Dual-Luciferase Reporter Assay System (Promega) was used to evaluate luciferase activity in accordance with the manufacturer’s instructions. The data were expressed as relative luciferase activity (firefly luciferase activity/renilla luciferase activity).

### 4.7. Dil-HDL Uptake Assay

The Dil-HDL binding assay was performed to evaluate cholesterol uptake [33]. After pretreatment with 10 μM PD98059 for 40 min following treated with the antibody, cells were incubated with 10 μg/mL Dil -HDL for an additional 4 h at 37 °C and observed in confocal microscopy (LSM780, Carl Zeiss, Oberkochen, BW, Germany). The analysis of Dil red fluorescence intensity was conducted on ZEN 3.4 software (Carl Zeiss).

### 4.8. Mice and Treatments

The ApoE^−/−^ mice were obtained from GemPharmatech (Nanjing, China) and were maintained on a 12 h light/dark cycle with the freedom to water. Male ApoE^−/−^ mice (4-week-old) were fed with HFD for 20 weeks and then transferred to a chow diet for one week, then the mice were injected intraperitoneally with different doses (0.5, 1.0, 1.5, 2.5, 3.0 mg) of the antibody or PBS [34]. The injections were repeated two times at 1-week intervals, and the mice were sacrificed two weeks after the last injection, and the livers were isolated to detect the expression of SR-BI, ApoA-I, ApoA-II, CYP7A1, and CYP27A1. All animal procedures were approved by the Animal Care and Use Committee of Southern Medical University and were performed in accordance with the National Institutes of Health Guidelines for the Care and Use of Laboratory Animals.

### 4.9. Ultrasound Imaging Analysis of Abdominal Aorta

The abdominal aorta diameter was determined by Vevo 2100 ultrasound imaging system (VisualSonics, Toronto, ON, Canada) as described previously [35]. Briefly, each mouse was anesthetized by isoflurane fixed on the heating platform, and then the hair on the abdomen was removed. The probe was placed transversely, just below the sternum and xiphoid process, and visualized the abdominal aorta with a color Doppler to confirm the abdominal aorta. Crop the ultrasound image to determine the blood vessel diameter and flow velocity.

### 4.10. Oil Red O Staining of Plaque

Aortas were dissected from mice with all adventitia removed. Aortas were then unfolded along the longitudinal axis, stained with Oil Red O, and photographed with a digital camera en face to examine the percentage of total atherosclerotic lesions in descending aorta (from the aortic arch to the bifurcation of the common iliac artery). The plaque area was analyzed by Image Pro Plus software.

### 4.11. Statistical Analysis

All data were obtained from at least three independent experiments and were analyzed via one-way ANOVA and the Student-Newman-Keuls (SNK) post hoc multiple comparison test by GraphPad Prism 6 software. The results are expressed as the mean ± standard deviation (SD). Results with *p* < 0.05 were considered statistically significant. *, *p* < 0.05; **, *p* < 0.01; ***, *p* < 0.001.

## Figures and Tables

**Table 1:** The content of total cholesterol, triglyceride, HDL-C, LDL-C in the liver (n = 7).

Group	Total Cholesterol (mmol/g)	Triglyceride (mmol/g)	HDL-C (mmol/g)	LDL-C (mmol/g)
PBS	0.053 ± 0.013	0.273 ± 0.131	0.341 ± 0.153	0.017 ± 0.005
6 Ab	0.050 ± 0.014	0.180 ± 0.025 *	0.552 ± 0.153	0.015 ± 0.003
14 Ab	0.048 ± 0.005	0.128 ± 0.017 **	0.811 ± 0.365 **	0.010 ± 0.007 *
6 Ab + 14 Ab	0.044 ± 0.007	0.153 ± 0.080 *	0.636 ± 0.208 *	0.011 ± 0.005 *

Data is presented as Mean ± SD; *, *p* < 0.05; **, *p* < 0.01.

**Table 2:** Changes of abdominal aorta diameter and blood velocity before and after administration.

Group	Pre-Treatment			Treatment		
	AA Diam (mm)	AA PSV (mm/s)	AA EDV (mm/s)	AA Diam (mm)	AA PSV (mm/s)	AA EDV (mm/s)
64 Ab-2.5 mg (n = 7)	0.809 ± 0.051	470.723 ± 41.270	40.841 ± 4.996	0.381 ± 0.055 **	237.897 ± 26.342 **	23.651 ± 1.483 **
14 Ab-2.5 mg (n = 9)	0.744 ± 0.021	416.076 ± 20.283	33.270 ± 6.050	0.536 ± 0.044 **#	253.507 ± 35.949 *	20.381 ± 3.205
14 Ab-3.0 mg (n = 8)	0.778 ± 0.043	457.699 ± 30.357	35.246 ± 3.727	0.498 ± 0.019 ***	247.004 ± 26.367 ***	27.266 ± 6.459

Data is presented as Mean ± SE. *, compared with Pre-treatment, *p* < 0.05; **, compared with Pre-treatment, *p* < 0.01; ***, compared with Pre-treatment, *p* < 0.001. #, compared with 64 Ab-2.5 mg (Treatment), *p* < 0.05. AA Diam: Abdominal Aorta diameter; AA PSV: Abdominal Aorta peak systolic velocity; AA EDV: Abdominal Aorta end diastolic velocity.

## Data Availability

The data obtained in this study are available in the article.

## Supplementary Materials

The following supporting information can be downloaded at: https://www.mdpi.com/article/10.3390/ijms232314607/s1.

## Abbreviations

RCT: reverse cholesterol transport; HDL, high-density lipoprotein; ABCA1, ATP-binding cassette sub-family A1; SR-BI, scavenger receptor class B type I; ApoA-I, apolipoprotein A-I; ApoA-II, apolipoprotein A-II; PPARα, peroxisome proliferator-activated receptor α; CYP7A1, cholesterol 7α-hydroxylase; CYP27A1, sterol 27-hydroxylase; FcRn, the neonatal Fc receptor.
