# Supplementary material for: Recombinant Humanized IgG1 Antibody Promotes Reverse Cholesterol Transport through FcRn-ERK1/2-PPARα Pathway in Hepatocytes
Source: Int J Mol Sci. 2022 Nov 23;23(23):14607. doi: 10.3390/ijms232314607 (PMC9736681; doi:10.3390/ijms232314607)
Supplement: Supplementary file 1 [file ijms-23-14607-s001.zip › ijms-1980937-supplementary.pdf]

Table S1 The qPCR primers of genes in this paper

Gene	Strand	Primer sequence (5′-3′)
Human SCARB1	sense	AATAAGCCCATGACCCTGAAGC
	antisense	GCCCCACATGATCTCACCC
Human ApoA-I	sense	CCCTGGGATCGAGTGAAGGA
	antisense	CTGGGACACATAGTCTCTGCC
Human ApoA-II	sense	CTGTGCTACTCCTCACCATCT
	antisense	CTCTCCACACATGGCTCCTTT
Human CYP7A1	sense	GAGAAGGCAAACGGGTGAAC
	antisense	GGATTGGCACCAAATTGCAGA
Human CYP27A1	sense	CGGCAACGGAGCTTAGAGG
	antisense	GGCATAGCCTTGAACGAACAG
Human FCGRT	sense	GGGGAAAAGGTCCCTACACTC
	antisense	CCTGCTTGAGGTCGAAATTCAT
Human GAPDH	sense	ACAACTTTGGTATCGTGGAAGG
	antisense	GCCATCACGCCACAGTTTC
Mouse SCARB1	sense	TTTGGAGTGGTAGTAAAAAGGGC
	antisense	TGACATCAGGGACTCAGAGTAG
Mouse ApoA-I	sense	GCTCAAGAGCAACCCTACCTT
	antisense	GCTTTCTCGCCAAGTGTCTTC
Mouse ApoA-II	sense	GCAGACGGACCGGATATGC
	antisense	GCTGCTCGTGTGTCTTCTCA
Mouse CYP7A1	sense	GCTGTGGTAGTGAGCTGTTG
	antisense	GTTGTCCAAAGGAGGTTCACC
Mouse CYP27A1	sense	GCACAGGAGAGTACGGAGG
	antisense	CGGGCAAGTGCAGCACATA
Human GAPDH	sense	AGGTCGGTGTGAACGGATTTG
	antisense	GGGGTCGTTGATGGCAACA
